# Spatial Engineering of Osteochondral Tissue Constructs Through Microfluidically Directed Differentiation of Mesenchymal Stem Cells

**DOI:** 10.1089/biores.2016.0005

**Published:** 2016-04-01

**Authors:** Stephen M. Goldman, Gilda A. Barabino

**Affiliations:** ^1^ Interdisciplinary Bioengineering Graduate Program, Georgia Institute of Technology, Atlanta, Georgia.; ^2^ G.W. Woodruff School of Mechanical Engineering, Georgia Institute of Technology, Atlanta, Georgia.; ^3^ Department of Biomedical Engineering, City College of New York, New York, New York.

**Keywords:** mesenchymal stem cells, microfluidic hydrogels, osteochondral repair, tissue engineering

## Abstract

The development of tissue engineered osteochondral units has been slowed by a number of technical hurdles associated with recapitulating their heterogeneous nature *ex vivo*. Subsequently, numerous approaches with respect to cell sourcing, scaffolding composition, and culture media formulation have been pursued, which have led to high variability in outcomes and ultimately the lack of a consensus bioprocessing strategy. As such, the objective of this study was to standardize the design process by focusing on differentially supporting formation of cartilaginous and bony matrix by a single cell source in a spatially controlled manner within a single material system. A cell-polymer solution of bovine mesenchymal stem cells and agarose was cast against micromolds of a serpentine network and stacked to produce tissue constructs containing two independent microfluidic networks. Constructs were fluidically connected to two controlled flow loops and supplied with independently tuned differentiation parameters for chondrogenic and osteogenic induction, respectively. Constructs receiving inductive media showed differential gene expression of both chondrogenic and osteogenic markers in opposite directions along the thickness of the construct that was recapitulated at the protein level with respect to collagens I, II, and X. A control group receiving noninductive media showed homogeneous expression of these biomarkers measured in lower concentrations at both the mRNA and protein level. This work represents an important step in the rational design of engineered osteochondral units through establishment of an enabling technology for further optimization of scaffolding formulations and bioprocessing conditions toward the production of commercially viable osteochondral tissue products.

## Introduction

The development of engineered tissue grafts has emerged as a promising therapeutic alternative for the repair and replacement of organs. A number of approaches, using a diverse spectrum of scaffolds, cell populations, and bioprocessing conditions, have been pursued for the production of such grafts. A plurality of these efforts has been centered around the development of homogenous tissues intended to mimic the functional properties of the target tissue *in vivo*. Some tissues, however, are heterogeneous both structurally and functionally and possess spatially varying biochemical compositions and mechanical properties for which the use of a single scaffolding material, cell source, or bioreactor chamber may be inappropriate. A classic example of this is the osteochondral unit, consisting of a hyaline cartilage layer and the integrated subchondral bone.

Osteochondral defects, resulting from traumatic injury, are typically treated through a grafting technique termed mosaicplasty.^2^ One of the primary shortcomings of mosaicplasty is the reliance on autologous graft sourcing from a healthy nonload bearing site that is both limited in its availability and potentially inappropriate for repair due to advanced osteoarthritic degeneration.^1,2^

To address this supply issue, a number of approaches have been pursued to create a suitable replacement for the autologous grafts. Common approaches to recapitulate the unique heterogeneity of the osteochondral unit include the production of composite scaffoldings loaded with one or more cell sources having chondrogenic and/or osteogenic potential and cultivating them utilizing both commercially available and custom-built bioreactor systems.^3^ Constructs produced in this manner, however, are still nonoptimal as they suffer from a number of shortcomings. Arguably, the most pertinent shortcoming of these approaches is their reliance on terminally differentiated cells (osteoblasts and chondrocytes) isolated from patient-specific biopsies and expanded *in vitro*. Use of terminally differentiated cells is plagued by the same dependency on an available autologous donor site, as well as low proliferation rates and potential degradation of functionality, should *in vitro* expansion be necessary to sufficiently populate the tissue engineered construct.^4^

Mitigation of this particular shortcoming can be accomplished by utilizing undifferentiated multipotent mesenchymal stem cells (MSCs) as a single autologous cell source for repair of osteochondral defects.^3,5–8^ MSCs are well-known progenitor cells for both the chondrocyte and osteoblast lineages, which have been used to generate osteochondral constructs using single-component or composite scaffolds across a range of compositions and material properties.^3,6,9–19^ The primary challenge to MSC-based constructs arises from the need to either utilize costly predifferentiation operations before the seeding of the construct or simultaneously modulate differentiation down to distinct lineages in a unified culture solution. Using conventional bioreactor systems, the popular approach of supplementing the culture media with lineage-specific signaling molecules to achieve directed differentiation of MSC is untenable for biphasic constructs without some means of spatially directed delivery to prevent dominance of one desired phenotype throughout the construct.^20^

Based on these realities, we hypothesized that the spatially confined presentation of optimized differentiation cues would result in tissue-specific inductive regions for the regeneration of both bone and cartilage tissues using a model universal donor cell source in an integrated tissue construct. To test this hypothesis, we utilized a microfluidic hydrogel platform previously developed in our laboratory to stimulate region-specific induction of osteoblastic and chondrogenic phenotypes through parallel, independent microfluidic networks and evaluated the constructs after 2 weeks of culture for the presence of differential gene expression and matrix composition between the osteogenic and chondrogenic layers.

## Materials and Methods

Supplies and reagents were obtained from VWR International, Sigma, or Invitrogen unless otherwise specified. Antibodies were from AbD Serotec or Abcam. ELISA kits for Collagens I and II were purchased from Chondrex, Inc. and for Collagen X from MyBioSource, Inc.

### MSC isolation and characterization

Bone marrow aspirates from bovine calves (Research 87) were mixed with expansion medium (high-glucose Dulbecco's modified Eagle's medium [DMEM] supplemented with 10% certified fetal bovine serum [FBS] and 1× penicillin–streptomycin–fungizone [PSF]) supplemented with 300 U/mL heparin and subjected to straining and centrifugation processes. Following centrifugation, pelleted cells were suspended in fresh expansion medium and plated onto T-75 flasks (Corning, Inc.). Nonadherent cells were removed from the flasks after 24 h, while adherent cells were cultured to confluence. Subsequent subculturing was carried out to Passage 3 at a splitting ratio of 1:3. Following Passage 3, MSCs were placed in a cryoprotective medium (70% DMEM, 20% FBS, 10% dimethyl sulfoxide [DMSO]) at a concentration of 1 million cells/mL stored in liquid nitrogen in 1 mL aliquots.

### Tissue culture

**FIG. 1.**
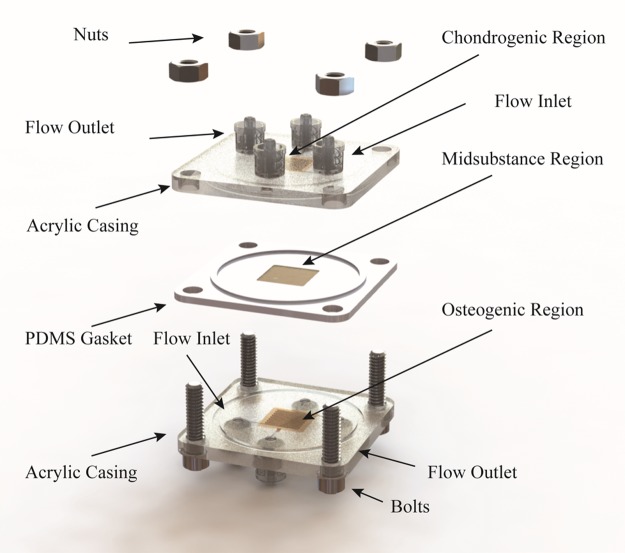
Construction process of the microfluidic osteochondral graft. Each target region is independently cast and controlled through ports in the acrylic casing. The chondrogenic and osteogenic regions are separated by a planar midsubstance region molded directly into a PDMS gasket, which ensures unidirectional flow through the microfluidic networks.

Constructs were fabricated by casting a cell-polymer solution (25 million MSC/mL; 2.5% agarose) against plasma-treated PDMS molds of the microfluidic network as described previously.^21^ Independent networks were formed by sealing a planar slab of the cell-polymer solution between the molded portions in an acrylic casing (Fig. 1). External flow loops were connected through luer-lock interfaces on the acrylic casing. Unidirectional flow of culture media was achieved using a syringe pump equipped with dual check valves. Capitalizing on the independence of the fluidic networks, culture commenced under regionally specific bioprocessing conditions.

Constructs from the experimental group received two different sets of bioprocessing conditions. The osteogenic region was provided with a serum-free basal media (high-glucose DMEM, 1× PSF, 0.1 μM dexamethasone, 50 μg/mL ascorbate 2-phosphate, 40 μg/mL l-proline, 100 μg/mL sodium pyruvate, 1× insulin–transferrin–selenium) supplemented with 10 ng/mL BMP-2 at a constant perfusion rate of 2.5 mL/min such that the shear stress distribution at the microchannel walls was a uniform 10 dyne/cm^2^. The chondrogenic region was supplied with serum-free basal media supplemented with 100 ng/mL TGF-β3 at 250 μL/min. The flow rate for the chondrogenic region was determined to both fulfill the minimal flow rate requirements for the nutrient demands of the resident cell population^21^ and to provide a uniform, low-magnitude shear stress distribution of 1 dyne/cm^2^ at the microchannel walls. For the control group, both microfluidic networks of the tissue constructs were provided with noninductive serum-free basal media at a flow rate of 250 μL/min flow rate. Total culture media volume was maintained at 100 mL with fresh media exchanges performed every 3–4 days. Gas exchange and pH balance were maintained by bubbling a 5% CO_2_ balance air gas mixture through the culture media reservoir.

### mRNA expression

Quantitative reverse transcription polymerase chain reaction (RT-qPCR) was used to quantify gene expression within the constructs in a region specific manner. RNA was isolated from the homogenized cell lysate according to the TRIzol protocol. Reverse transcription of the RNA into cDNA was achieved using a QuantiTect Rev Transcription Kit (QIAGEN). Real-Time PCR amplification was performed (StepOnePlus™; Applied Biosystems) in the presence of SYBR Green/ROX master mix and primers for target osteochondral lineage markers (Supplementary Table S1). Regulation of the target genes over day 0 controls was determined by processing the raw fluorescence data using LinRegPCR (v12.11; www.hartfaalcentrum.nl) with glyceraldehyde-3-phosphate dehydrogenase (*GAPDH*) and β-actin (*ACTB*) serving as the endogenous controls through geometric averaging.^22^

### Biochemical analyses

Construct weights (wet weight) were taken before freezing (−80°C) and subsequent lyophilization. Lyophilized samples were weighed again (dry weight) and digested in papain buffer for 16 h at 60°C. Aliquots of digested samples were assessed for DNA content using a PicoGreen dsDNA Kit. Glycosaminoglycan (GAG) content was measured using the dimethylmethylene blue dye-binding assay.^23^ Quantification of collagen types I, II, and X was carried out using ELISA Kits as per manufacturers' protocols.

### Histological analyses

Tissue constructs were fixed in 10% (v/v) neutral-buffered formalin, dehydrated through an ethanol gradient, embedded in paraffin wax, and cut into sections of 8.0 μm. Deparaffinized sections were then stained with Toluidine Blue for proteoglycans and Alizarin Red for calcium. For immunofluorescence, samples were blocked for 30 min and incubated with primary rabbit antibodies (1:100) against collagen types I, II, and X at 4°C overnight following antigen retrieval using the citrate buffer method. Sections were then washed thrice in PBS and treated with goat anti-rabbit secondary antibodies (1:200) for 1 h at room temperature. Sections were washed once more and mounted with VECTASHIELD containing DAPI. Photomicrographs were captured on an inverted fluorescence microscope (Nikon Instruments, Inc.) equipped with a CCD camera (CoolSNAP HQ2 CCD; Photometrics).

### Statistical analysis

Sample sizes for RT-qPCR and biochemical analyses were *n* = 3 and *n* = 5, respectively. Bar graphs are presented as the mean ± SEM with statistically significant differences defined as *p* < 0.05 using two-way ANOVA with Bonferroni *post hoc* tests for multiple comparisons.

## Results

### Cellular content

**FIG. 2.**
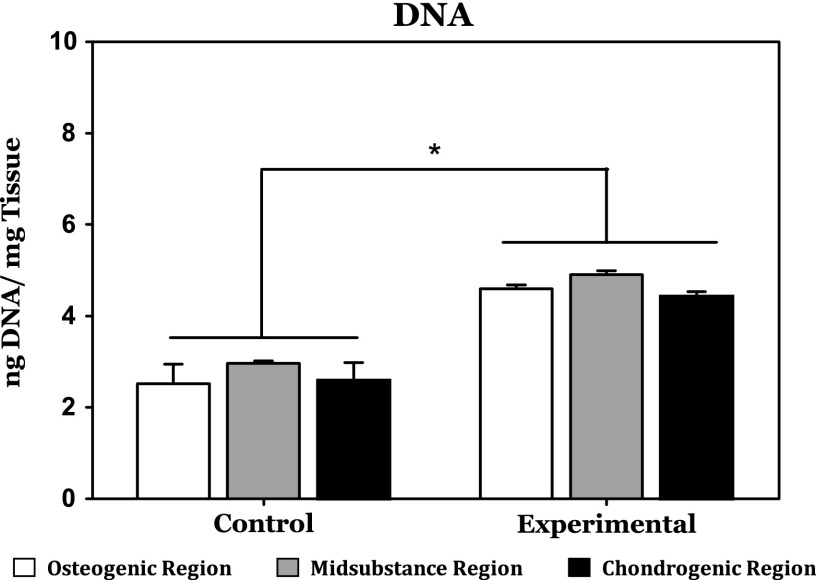
After 2 weeks of culture, DNA content was significantly higher in the experimental group, which received cytokine supplementation relative to the unsupplemented control group. There were no significant differences between the various regions of the experimental cultures. *Indicates a statistically significant finding (*p* < 0.05).

As depicted in Figure 2, there was no significant difference in DNA content between any of the spatially distinct regions within either the experimental or control groups. There was, however, a statistically significant difference in DNA content between the control and experimental groups across all phenotypic regions of the tissue constructs after 2 weeks of culture. This difference indicates increased cell proliferation within the experimental group relative to the control group.

### Differential expression of osteochondral genes

**FIG. 3.**
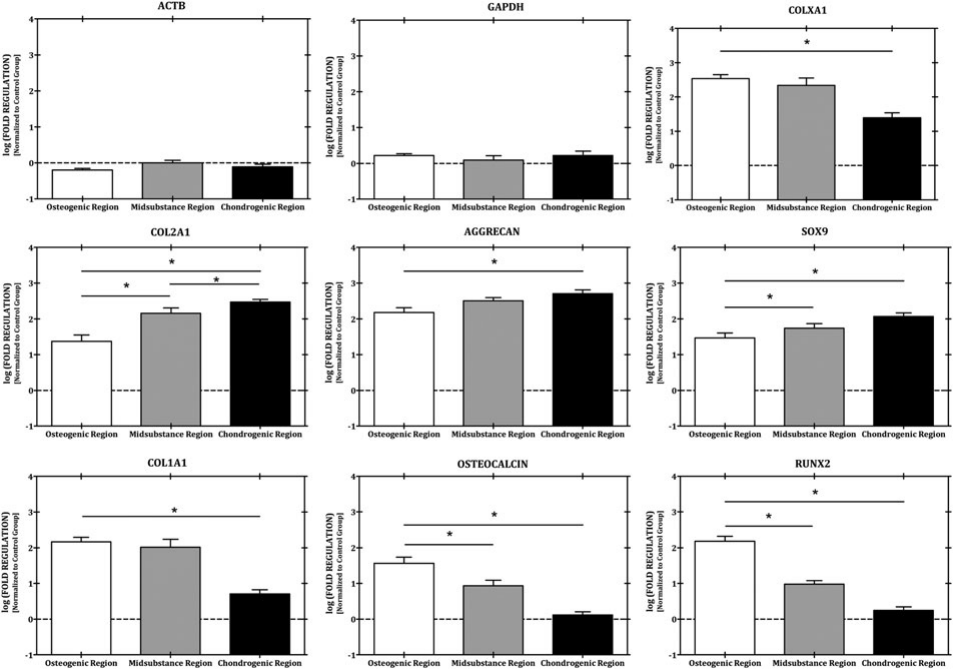
Differential loading of an osteochondral tissue construct results in gene expression gradients of both osteogenic and chondrogenic genes. *Indicates a statistically significant finding (*p* < 0.05).

Within the control group, no difference was observed in the osteogenic (*RUNX2, OSTEOCALCIN,* and *COL1A1*), hypertrophic (*COLXA1*), or chondrogenic (*SOX9, AGGRECAN,* and *COL2A1*) gene expression profiles between the various regions of the tissue constructs. Within the experimental group, however, differential expression of both the osteogenic and chondrogenic gene expression profiles with respect to the opposing construct region was observed (Fig. 3). Within the osteogenic target region, a statistically significant upregulation of *RUNX2* (86-fold) and *COL1A1* (29-fold) was observed relative to the chondrogenic target region. Regulation of the osteogenic gene panel was also greater compared with the chondrogenic panel with the exception of aggrecan, but not in a statistically significant manner. With regard to the chondrogenic gene panel, a statistically significant regulation of the entire chondrogenic gene panel (*SOX9, AGGRECAN,* and *COL2A1*) within the chondrogenic region was observed relative to the osteogenic target region of the construct. In addition, *COLXA1* expression was observed to increase across the construct from the chondrogenic regions to the osteogenic region, with a statistically significant difference in expression occurring between the chondrogenic and osteogenic regions, but not with such difference occurring between the midsubstance and osteogenic regions.

### GAG content

**FIG. 4.**
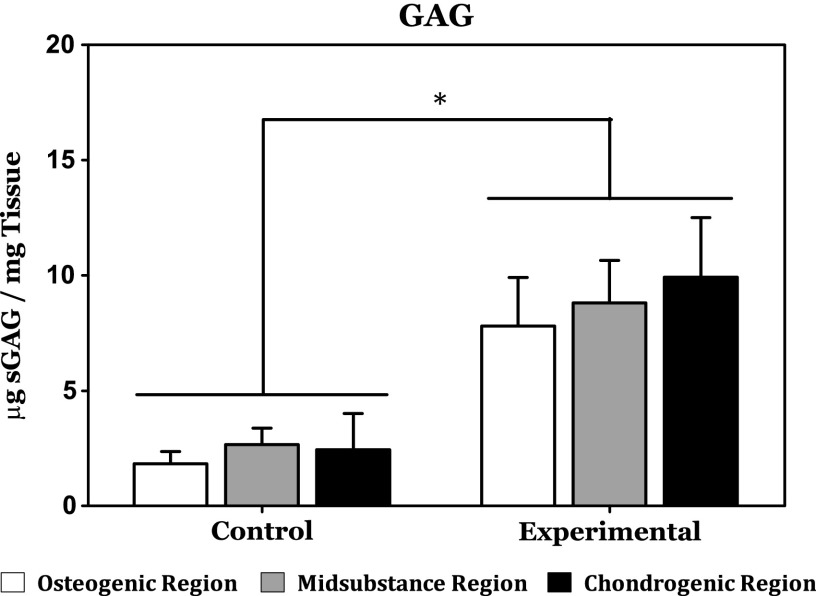
Measurement of sulfated glycosaminoglycan content within the various regions of the osteochondral constructs by DMMB assay reveals significantly higher GAG accumulation in the experimental group irrespective of the construct region relative to the control group. Within the experimental group, however, no statistically significant differences were observed. GAG, glycosaminoglycan. *Indicates a statistically significant finding (*p* < 0.05).

As evidenced by the results of the DMMB assay, GAG content was significantly higher in the experimental group relative to the control group (Fig. 4). In addition, within the experimental group, GAG content was on average highest in the chondrogenic target region and lowest in the osteogenic region. The difference in average GAG content in the chondrogenic and the osteogenic regions of the experimental group, however, was not considered statistically significant. In addition, no statistically significant difference in GAG content was observed between the construct regions of the control groups.

### Graded collagen expression

**FIG. 5.**
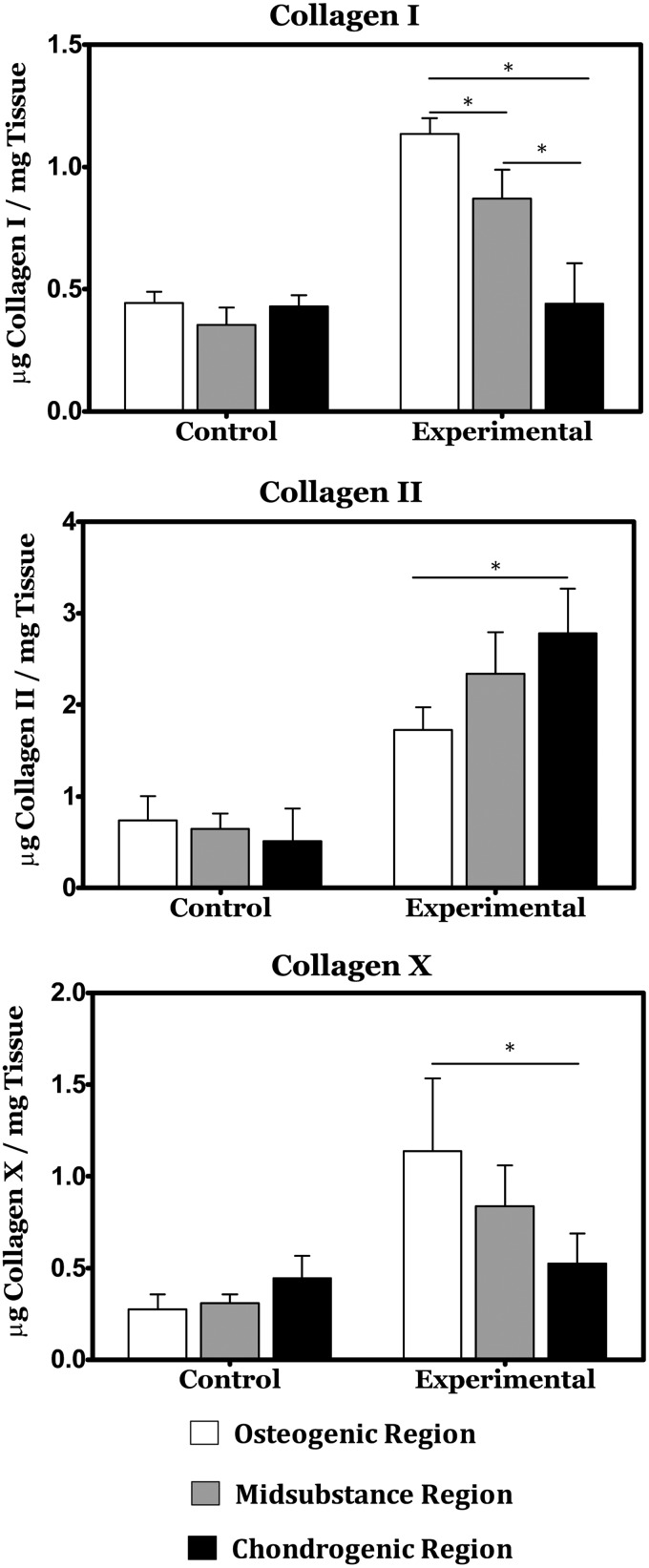
ELISA reveals gradients in Collagen type I across the osteochondral constructs and differential expression of collagen types II and X between the osteogenic and chondrogenic regions of the experimental group. *Indicates a statistically significant finding (*p* < 0.05).

ELISA was performed for expression of collagens Type I, II, and X. As depicted in Figure 5, all three collagen types exhibited graded expression across the construct thickness, with types I and X exhibiting their maximum concentration in the osteogenic target region of the construct and type II exhibiting a maximum concentration in the chondrogenic region of the construct. Within the control group, collagen content was significantly lower with no gradations of note.

### Histology and immunofluorescence

**FIG. 6.**
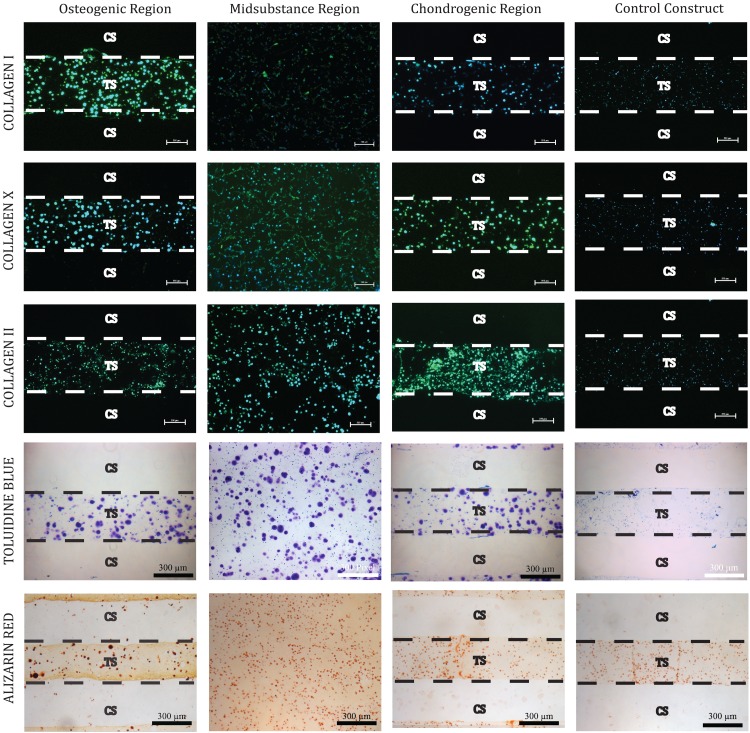
Immunofluorescence staining shows gradients in collagen types I and II. Alizarin red staining also indicates increased mineralization within the osteogenic region relative to the chondrogenic region.

Control constructs stained weakly and relatively homogeneously for both histological stains and for all collagens tested following 2 weeks of culture (Fig. 6). The experimental group, however, exhibited much stronger staining across all regions. Within the experimental group, Toluidine Blue staining revealed no discernible difference in proteoglycan content between the various regions of the osteochondral constructs. Alizarin Red staining revealed a slight gradient in mineralization with a region of high concentration within the osteogenic layer and a region of low concentration in the chondrogenic layer. Collagen staining revealed a mild gradient in both type I and type II collagen with the highest concentration of each located within the osteogenic and chondrogenic layers, respectively. Collagen X staining results were inconclusive between regions in the experimental group, but clearly higher relative to the control group.

## Discussion

The purpose of the study described herein was to evaluate microfluidic hydrogels as a platform for the production of osteochondral tissue constructs through the spatially directed differentiation of bovine MSCs. The ability of the mechanochemical inductive cues provided through the microfluidic networks to direct targeted phenotype induction was evaluated through gene expression analysis, biochemical composition, and histological staining. Relative to our noninductive control cultures, the spatially defined presentation of inductive factors and bioprocessing conditions had a clear impact in proliferation of the resident cell population and elaboration of a spatially discrete osteochondral matrix within our experimental group. On a whole construct basis, differences between the control and experimental constructs included significant increases in both DNA content and total osteochondral matrix elaboration. These findings are in agreement with the prior literature on the effects of the TGF-β superfamily proteins provided to these cultures,^24,25^ as well as to previous findings from our group on the synergistic effects of hydrodynamic loading on MSC differentiation efficiency in the presence of these factors.^26^ Within the experimental group, there was evidence of spatial differences in matrix composition reminiscent of the osteochondral junction. The chondrogenic target region of the construct showed a local maximum of GAG content and significantly higher expression of Collagen II relative to the osteogenic target region, while significantly higher expression of Collagen I and X was observed in addition to a minimum in GAG content in the osteogenically targeted region of the constructs. In addition, Alizarin Red staining showed an increase in mineralization within the osteogenic region. The dual presence of GAG and mineralization within the osteogenic region indicates the concurrent formation of both cartilage and bone, and may represent an intermediate differentiation step along the endochondral ossification pathway for the culture period studied herein, rather than a terminal bone phenotype. While suboptimal, we believe this result to be acceptable for the culture durations studied and hypothesize that cultivation for longer durations would result in replacement of the cartilaginous portion of the matrix with higher quality bone formation.^25,27^ This is further supported by the relatively lower presence of both Collagen I and mineralization in the chondrogenic region indicating that this endochondral bone formation is concentrated near the BMP-2 supply network and that the cartilage formation in the chondrogenic region is hyaline in character.

In benchmarking our technology in the context of other studies, we note that a number of studies have been reported utilizing dual culture control systems for osteochondral tissue engineering. Chang et al. cultured a gelatin-infused sinbone block to generate osteochondral constructs in a dual-chambered bioreactor approach that validated their scaffolding system for the production of hyaline cartilage within the gelatin portion of the composite scaffolding.^28^ The bony portion of this scaffold, however, was acellular in nature. Mahmoudifar and Doran used a similar dual-chambered bioreactor to that implemented by Chang et al. for the production of osteochondral tissue constructs from two sutured together polyglycolic acid meshes seeded with adipose-derived stem cells.^29^ This approach mirrored our results after 2 weeks of culture with respect to statistically indeterminate differences in GAG content between the layers, but was not in agreement with our finding of differential expression of collagen II. Compared to these studies, our constructs are not only cellularized in both the osteogenic and chondrogenic regions as was also shown by Mahmoudifar and Doran but also our system was shown to suppress osteogenic character within the chondrogenic layer. While the characteristics of the cell type seeded in each of these systems may also play a role in the improvement shown with respect to this metric, we believe the improvement is due to improved control of the microenvironment provided by the presence of the microfluidic network within the tissue construct versus the superficial delivery of inductive cues characteristic of the dual-chambered bioreactor. While more involved than the dual-chambered bioreactor, the paradigm proposed by our system offers the capability to produce thicker constructs as necessary and even greater opportunities for optimization of culture conditions through the incorporation of independent microfluidic networks into the construct.

## Conclusions

In this study, we have established a paradigm for the production of biphasic tissue constructs through microfluidically directed differentiation of MSCs using the osteochondral unit as a model tissue. While there is evidence in the literature of other approaches to spatially engineer the composition of an osteochondral construct, this study is the first of its kind to utilize microfluidic networks to successfully engineer a biphasic tissue of clinically relevant thickness with measurable differences in biochemical composition between the bony and cartilaginous regions. The results presented herein highlight how an optimized mechanochemical microenvironment can affect the production of tissue-specific extracellular matrix of the resident cell population seeded in the various regions of a hydrodynamically loaded osteochondral construct compared to control constructs produced through a noninductive bioprocessing scheme. Based on our results, we believe that this approach may have significant potential for the production of the osteochondral unit, as well as other interfacial tissues for use in regenerative capacities. We would be remise, however, if we did not address the dependency of the ultimate utility of this approach on the further development of enabling material and biofabrication technologies to help achieve cost-effective production and processing of well-defined robust tissue products. The promising findings of the present study represent an important first step in the rational design of engineered osteochondral units through establishment of a platform for the future optimization of scaffolding formulations and bioprocessing parameters toward the production of commercially viable osteochondral tissue products using microfluidic scaffolding strategies.^30^

## Supplementary Material

Supplemental data

## Abbreviations Used

DMEM: Dulbecco's modified Eagle's medium
DMSO: dimethyl sulfoxide
FBS: fetal bovine serum
GAG: glycosaminoglycan
GAPDH: glyceraldehyde-3-phosphate dehydrogenase
MSC: mesenchymal stem cell
PSF: penicillin–streptomycin–fungizone
RT-qPCR: quantitative reverse transcription polymerase chain reaction
