# Supplementary material for: Spatial Engineering of Osteochondral Tissue Constructs Through Microfluidically Directed Differentiation of Mesenchymal Stem Cells
Source: Biores Open Access. 2016 Apr 1;5(1):109–17. doi: 10.1089/biores.2016.0005 (PMC4854211; doi:10.1089/biores.2016.0005)
Supplement: Supplemental data [file Supp_Table.pdf]

Supplementary Data

Supplementary Table S1. RT-qPCR Primers

Function	Gene	Primer nucleotide sequence
Housekeeping genes	ACTB	Forward 5′GAGCGGGAAATCGTCC GTGAC 3′
		Reverse 5′ GTGTTGGCGTAGAGGTC CTTGC 3′
	GAPDH	Forward 5′ CCTTCATTGACCTTCACT ACATGGTCTA 3′
		Reverse 5′ TGGAAGATGGTGATGGC CTTTCCATTG 3′
Chondrogenic markers	sox9	Forward 5′ CATGAAGATGACCGACG AG 3′
		Reverse 5′ CGTCTTCTCCGTGTCGGA 3′
	Aggrecan	Forward 5′ CACTGTTACCGCCACTT CCC 3′
		Reverse 5′ GACATCGTTCCACTCGC CCT 3′
	col2α1	Forward 5′ ATCCATTGCAAACCCAA AGG 3′
		Reverse 5′ CCAGTTCAGGTCTCTTAG AG 3′
Hypertrophic marker	colXα1	Forward 5′ CATGCTGCCACAAACAGC 3′
		Reverse 5′ TGGATGGTGGGCCTTTTA 3′
Osteogenic markers	runx2	Forward 5′ TTACAGACCCCAGGCA GGCACA 3′
		Reverse 5′ TCCATCAGCGTCAACAC CATCA 3′
	Osteocalcin	Forward 5′ TGACAGACACACCATGA GAACCC 3′
		Reverse 5′ AGCTCTAGACTGGGCCGT AGAAG 3′
	col1α1	Forward 5′ TGCTGGCCAACCATGCC TCT 3′
		Reverse 5′ CGACATCATTGGATCCTT GCA G 3′

ACTB, β-actin; GAPDH, glyceraldehyde-3-phosphate dehydrogenase; RT-qPCR, quantitative reverse transcription polymerase chain reaction.
